# Application of exogenous electron mediator in fermentation to enhance the production of value-added products

**DOI:** 10.1128/aem.00495-25

**Published:** 2025-05-12

**Authors:** Yingxuan Yu, Zhongliang Shi, Weiming Li, Mengyang Bian, Chi Cheng, Yimei Xi, Shuhua Yao, Xiangfeng Zeng, Yongfeng Jia

**Affiliations:** 1 Liaoning Engineering Research Center for Treatment and Recycling of Industrially Discharged Heavy Metals, Shenyang University of Chemical Technology 118412, Shenyang, China; 2 Key Laboratory of Pollution Ecology and Environmental Engineering, Institute of Applied Ecology, Chinese Academy of Sciences 74763 , Shenyang, China; 3 MOE Key Laboratory of Bio-Intelligent Manufacturing, Engineering Research Center of Application and Transformation for Synthetic Biology, School of Bioengineering, Dalian University of Technology 592991, Dalian, China; 4 Ningbo Institute of Dalian University of Technology 659738, Ningbo, China; University of Illinois Urbana-Champaign, Urbana, Illinois, USA

**Keywords:** exogenous electron mediator, fermentation, value-added products, electron transfer, metabolic network

## Abstract

Electron transfer is essential for the production efficiency of value-added products in anaerobic fermentation, such as butanol and ethanol as biofuels, and short-chain fatty acids (SCFAs) including butyric acid and acetic acid as platform chemicals. Electron mediators (EMs), also known as electron shuttles, can facilitate electron transfer to counter irreversible or slow redox reactions that limit fermentation. The addition of EMs has been shown to be an effective strategy to promote fermentation by various bacteria, particularly *Clostridium* species, for these valuable product syntheses. This paper reviews recent advancements in the application of exogenous electron mediators (EEMs) across various scenarios. Common EEM types, their characteristics, and mechanisms are summarized, and different application scenarios are discussed to elucidate the effect of EEMs. Key technical challenges and future directions for EEM application are also explored.

## INTRODUCTION

**Fig 1:**
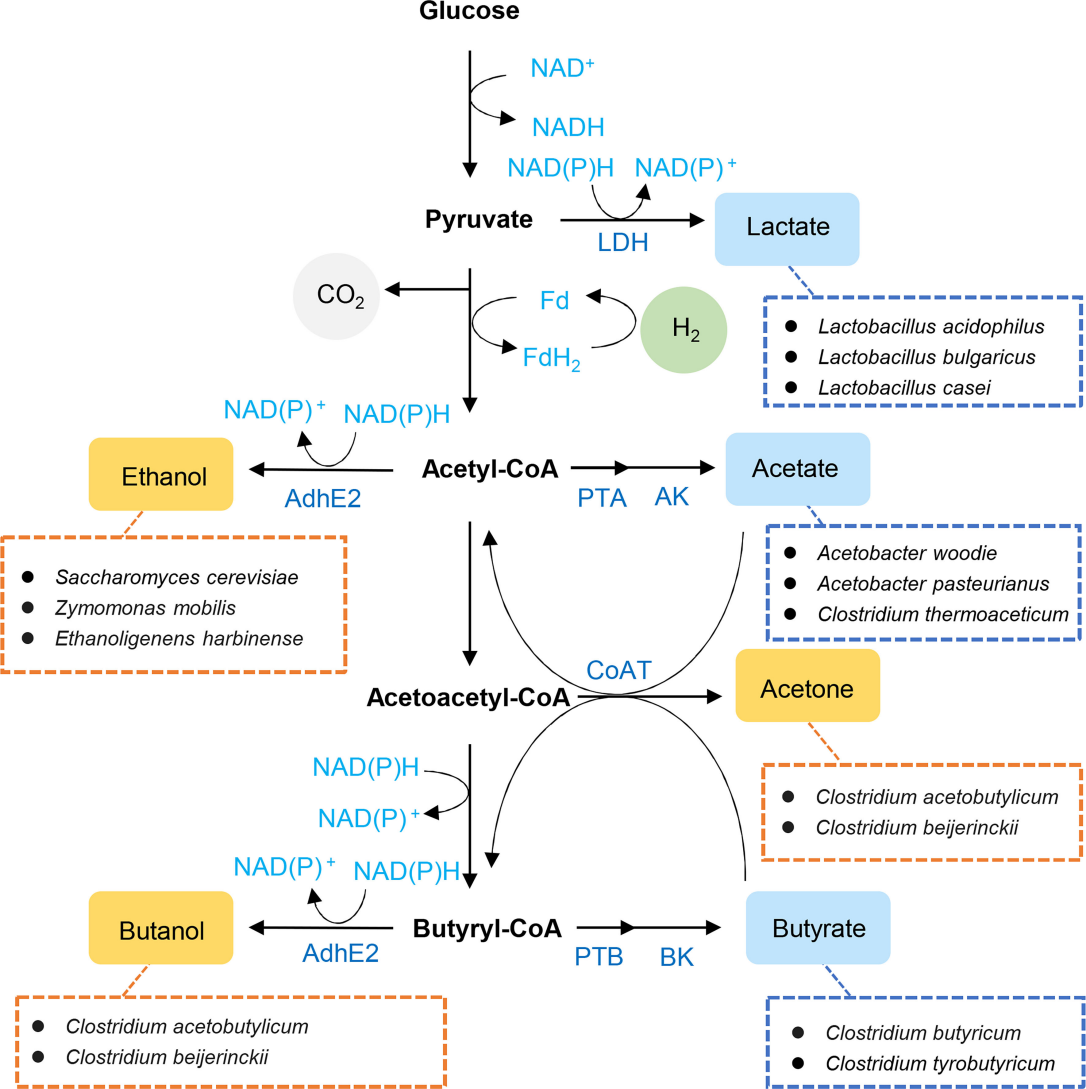
Core metabolic pathways of anaerobic fermentation. Yellow box: solvent products; blue box: acid products. AdhE2: aldehyde/alcohol dehydrogenase; AK: acetate kinase, BK: butyrate kinase; CoAT: CoA transferase; LDH: lactate dehydrogenase; PTA: phosphotransacetylase; PTB: phosphotransbutyrylase. Representative strains are shown for each product category; additional species may contribute to similar pathways.

Abundant organic wastes are potential resources to recover chemicals, nutrients, and fuels (1). Resource recovery by microorganisms is considered carbon-neutral due to its biogenic nature, gaining immense attention in the circular economy framework (2). Anaerobic fermentation is a biological process in which anaerobic or facultatively anaerobic microorganisms extract energy by degrading organic matter in the absence of oxygen (3). These bacteria can utilize various feedstocks including wastewater, lignocellulosic biomass, food wastes, and industrial wastes, producing short-chain fatty acids (SCFAs), solvents, and hydrogen (Fig. 1), with specific strains demonstrating unique production capabilities. For instance, *Clostridium acetobutylicum* is known for efficient acetone-butanol-ethanol production, whereas *Clostridium tyrobutyricum* and *Clostridium butyricum* excel in butyric acid synthesis and hydrogen evolution (4–6). The combination of versatile bacterial strains, diverse feedstock availability, and broad product spectrum makes anaerobic fermentation widely applicable in environmental and industrial fields, including organic waste and wastewater treatment as well as biofuel and value-added product generation (7, 8).

In anaerobic fermentation, carbohydrates are degraded via the glycolysis pathway, generating pyruvate, ATP, and NADH (Fig. 1). NADH is a crucial native electron carrier or mediator in biochemical reactions. Pyruvate is further broken down through various pathways to form different end metabolites (9). In one pathway, pyruvate can be directly reduced to lactate with NADH oxidation. Alternatively, pyruvate undergoes decarboxylation to form acetyl-CoA, which serves as a key intermediate for various products including acetate, propionate, butyrate, ethanol, and butanol through specific enzymatic pathways. During these conversions, oxidized nicotinamide adenine dinucleotide (NAD^+^) is regenerated through NADH oxidation in the formation of most end products except acetate. Besides NADH, ferredoxin (Fd), produced during pyruvate decarboxylation, serves as another crucial intracellular electron carrier (Fig. 1). Reduced Fd (Fd_red_) can either donate electrons to NAD^+^ via ferredoxin:NAD^+^ oxidoreductase (FNOR) to form NADH or transfer electrons to protons via hydrogenase to form molecular hydrogen. These electron transfer processes, mediated by intracellular carriers, play a fundamental role in determining the distribution of fermentation products.

Given their requirement for electrons, increasing electron supply is an effective approach to enhance product yields. Various strategies have been developed for electron enrichment in fermentation. For example, the addition of exogenous electron donors, such as nano zero-valent iron and biochar, can alter the electron quantity (10) or electron distribution between products (6, 10, 11). Direct supply of electrons through electrodes in electro-fermentation (EF) has also been demonstrated as an effective approach (12). Metabolic engineering has also been applied to increase intracellular reducing power (13).

Beyond these electron-enrichment strategies, manipulating the electron transfer is also an effective way to enhance fermentation. Electron mediators (EMs) are substances that can accept and transfer electrons during electron transfer processes (14). They can accept electrons from cells, transitioning from an oxidized state to a reduced state, and subsequently transfer these electrons to an electron acceptor, returning to their oxidized state. These mediators serve as bridges in biological redox reactions and facilitate electron transport both within and outside the cell. Microbes have native EMs, such as NADH, Fd, riboflavin, coenzyme Q, and phenazine derivatives (15–22). The quantity or efficiency of native EMs usually cannot meet the requirements for efficient synthesis of value-added products in fermentation, necessitating the introduction of EEMs (23).

The application of EEMs in fermentation processes holds significant potential for enhanced production of high-value products; however, there remains a notable lack of systematic reviews on EEMs, hindering insight into their current status and future development. This review aims to address this gap by providing a comprehensive overview of the classification and various applications of EEMs while summarizing their underlying mechanisms. By elucidating these aspects, we seek to highlight the importance and prospects of EMs in enhancing fermentation efficiency and product yield.

## CLASSIFICATION AND CHARACTERISTICS OF EEMs

Based on their origins, EEMs can be classified into biogenic and artificial mediators. Biogenic EMs, such as humic substances (HS), are redox-active substances synthesized by cells that possess specific physiological functions. These EMs can be extracted and supplemented into fermentation systems to increase fermentation production. Artificial EMs, on the other hand, are redox-active substances that cells cannot synthesize on their own. Key examples include viologen compounds, neutral red (NR), quinone compounds, and biochar (24–26).

### Viologen compounds

Viologens refer to a class of *N*, *N*'-disubstituted-4,4'-bipyridine salts, first recognized for their reduction reactions and electron-accepting properties by Michaelis et al. in 1933 (27). Viologens exist in divalent cationic, radical cationic, and neutral states (28). As typical electron-deficient organic ligands, viologens demonstrate excellent electron-accepting capabilities, making them a key class of redox-active compounds. Historically employed as herbicides, they have subsequently gained prominence as redox catalysts in biological systems. Among the viologen family, methyl viologen (MV) and benzyl viologen (BV) represent the most frequently utilized EEMs in anaerobic fermentation processes (29, 30).

**Fig 2:**
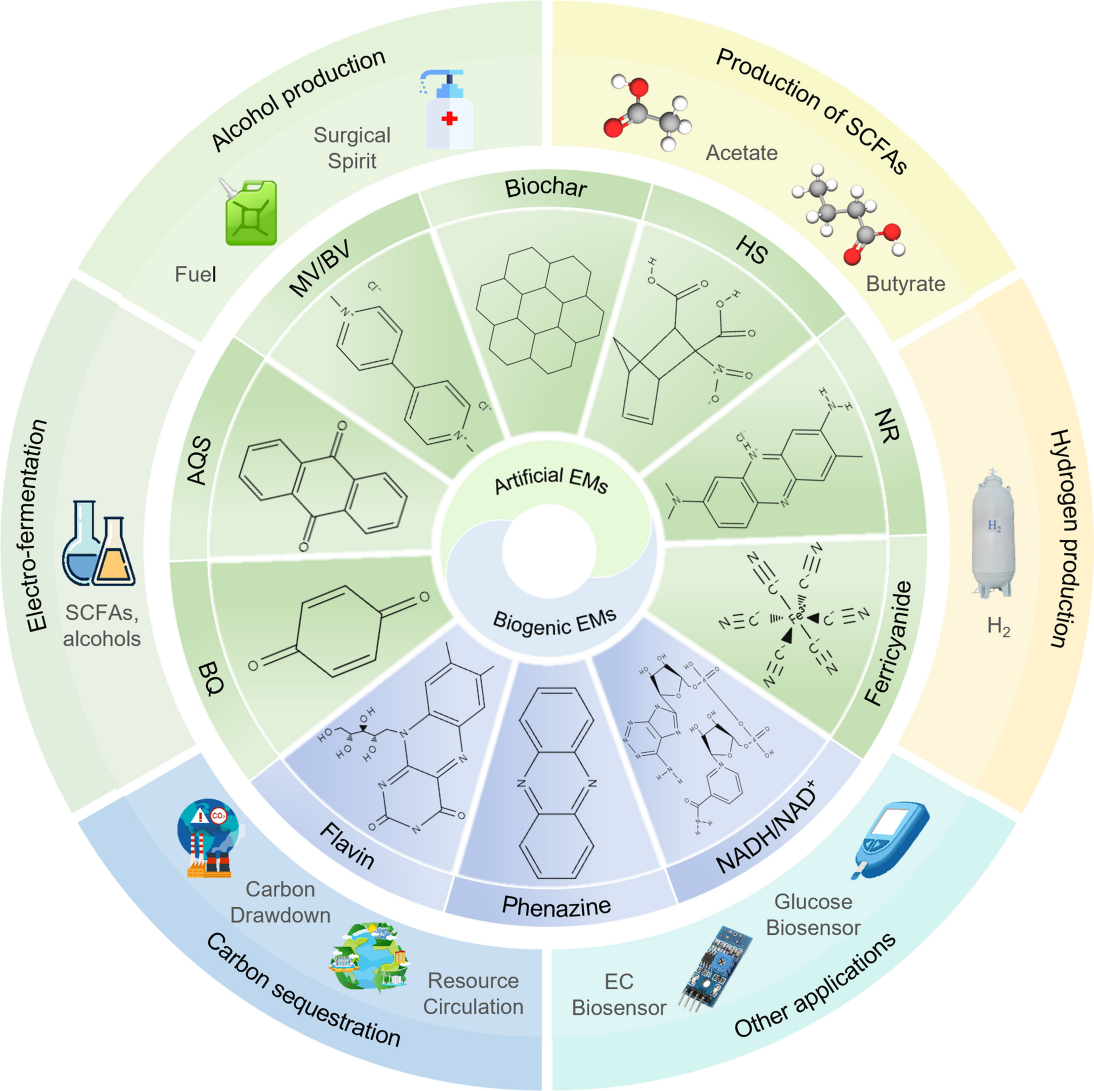
Classification, structures, and diverse applications of EEMs.

MV, also known as paraquat, mediates electron transfer through its bipyridinium core structure, where nitrogen atoms reversibly accept and donate electrons, switching between three redox states: MV^2+^, MV^+^, and MV^0^ (Fig. 2). The low potential of MV enables it to substitute Fd, the direct electron donor for hydrogenase in anaerobic fermentation (31). However, MV has also been shown to be toxic, inhibiting cell growth (32).

BV is another common viologen EEM with a 4,4'-bipyridinium redox-active core, distinguished by its benzyl substituents. BV offers advantages in terms of synthesis simplicity and, analogous to MV, can exist in three redox states. Similar to MV, BV influences anaerobic fermentation by reducing hydrogen production and enhancing NADH availability (30). This electron flow modulation capability makes BV an effective mediator for optimizing the production of value-added fermentation products that require NADH as a cofactor.

### Neutral red

NR, or 3-amino-7-dimethylamino-2-methylphenazine hydrochloride, is a cationic dye characterized by low toxicity and low standard reduction potential (33). The electron-mediating capacity of NR is attributed to its phenazine ring structure, which can readily switch between oxidized (NR^+^) and reduced (NRH₂) states (Fig. 2; Fig. S1). The reduced form, NRH₂, can influence bacterial metabolism by reducing NAD^+^ to NADH. NR interacts with bacteria by accepting electrons from hydrogenase and transferring them to dissolved iron or the anode in microbial fuel cells (34, 35).

### Humic substances

HS, dark-colored polydisperse organics formed during organic residue degradation, are classified based on pH-dependent solubility into humin (HM), humic acid (HA), and fulvic acid (FA) (36, 37). The electron-mediating capacity of HS derives primarily from their quinone moieties (C = O) that accept electrons to form hydroquinones and phenolic hydroxyl groups (C-OH) that can donate electrons (Fig. 2; Fig. S1). In anaerobic fermentation, HS serves as both electron sources and electron acceptors, with electrons potentially transferred through membrane proteins to reduce NAD^+^ and oxidized Fd (Fd_ox_) (38).

### Biochar

Biochar, a carbon-rich material produced through the thermochemical conversion of biomass, exhibits properties such as mild alkalinity, high porosity, and abundant surface functional groups (6, 39). Among these functional groups, oxygen-containing species are primarily responsible for the redox activity of biochar (40). For instance, phenolic groups act as electron donors, whereas quinones serve as electron acceptors, contributing to the electron-mediating capacity of biochar (41). These redox properties, combined with other physicochemical characteristics, enable biochar to facilitate anaerobic fermentation for various value-added products (6, 42, 43). Compared with other EEMs, biochar possesses advantageous properties such as low cost and resource abundance. It can be produced from various organic wastes, such as food waste, lignocellulosic biomass, feces, and sludge (44). Additionally, the associated by-products enhance the cost-effectiveness of biochar compared with conventional EEMs.

## APPLICATION OF EEMs IN DIFFERENT SCENARIOS

The applications of EEMs in fermentation processes have demonstrated significant potential across various scenarios. Different types of EEMs have been successfully employed to enhance the production of valuable products such as biofuels, organic acids, and hydrogen. Understanding how these diverse mediators perform in specific production scenarios provides valuable insights into their effectiveness and potential for industrial implementation. The following sections discuss specific applications of EEMs in different fermentation processes, highlighting their effects on product yields and process efficiency.

### Alcohol production in anaerobic fermentation

Alcohols are versatile chemicals used as fuels, solvents, and intermediates in chemical synthesis. *Saccharomyces cerevisiae*, *Zymomonas species*, and *Clostridium* species are widely used for alcohol production. *C. acetobutylicum* and *Clostridium beijerinckii* are the representative organisms for acetone-butanol-ethanol (ABE) fermentation due to their robust butanol-producing capacity (4). In these strains, aldehyde/alcohol dehydrogenase (AdhE2) catalyzes alcohol synthesis after pyruvate decarboxylation (Fig. 1). During the acidogenesis phase of anaerobic fermentation, adding EMs is an effective strategy to enhance alcohol production (Table 1).

**TABLE 1:** Main application of EEM in anaerobic fermentation

Classification	Electronic mediator	Bacterial strain	Function description	Reference
Alcohol production	MV	*C. tyrobutyricum* Δ*ack–adhE2*	Butanol production increased by 40%	(45)
		*C. acetobutylicum*	Butanol yield increased	(46)
		*Clostridium* sp. BC1	Ethanol production increased 28 times, butanol production increased 12 times	(47)
		*C. butyricum*	1,3-propanediol production increased	(48)
	BV	*C. acetobutylicum* YM1	Butanol productivity increased	(49)
	NR	*C. pasteurianum* DSM	Butanol yield increased by 33%	(50)
	biochar	*C. carboxidivorans*	Both ethanol and butanol production increased, and butanol production increased by four times	(51)
		*C. beijerinckii* F-6	Butanol yield increased by 20.23%	(52)
SCFAs production	NR	*Methylobacterium extorquens*	Reduction of CO_2_ to formic acid	(53)
		*C. tyrobutyricum* BAS 7	Butyric acid production increased	(5)
	BV	*C. tyrobutyricum*	The yield of butyric acid was increased by 50%, and the formation of acetic acid was inhibited	(30)
	HS	*/*	The production of carboxylic acids and H_2_ is increased	(54)
Hydrogen production	AH_2_QDS	*C. beijerinckii* NCIMB 8052	The hydrogen yield per mole increased by 24-37%	(55)
	AQS	*/*	The hydrogen yield increased by 11.4%	(56)
		*/*	Biohydrogen yield was remarkably enhanced to 24.9 mL/g VSS	(57)
	biochar	*E. aerogenes* and *E. coli*	The cumulative hydrogen production increased by four times	(58)
Electro-fermentation	NR	*C. autoethangenum*	The production of acetate and lactate increased	(59)
		*C. pasteurianum*	The yield of n-butanol increased by 33%	(50)
		*C. beijerinckii* NCIMB 8052	Butanol yield increased by 172.7%	(60)
	MV	*C. acetobutylicum*	Butanol yield increased by 56%	(61)
		*Clostridia spp*	The yield of butanol is increased, and hydrogen generation is inhibited	(62)
Carbon sequestration	MV, biochar	*C. carboxidivorans*	Carbon fixation, promoted the synthesis of butyric acid and caproic acid	(29)

Viologens (MV and BV) generally show the best performance, with BV being more effective at lower concentrations compared with MV (29). The addition of MV to *C. acetobutylicum* fermentation was studied as early as 1987, and it was found that MV significantly increased butanol production, with a positive correlation between MV concentration and butanol yield (46).

Similarly, the addition of BV led to an increase in butanol productivity butanol productivity and a higher butanol-to-acetone ratio (49). In *C. tyrobutyricum* Δ*ack–adhE2* fermentation, the combination of MV addition and genetic engineering raised butanol yield by 40% while reducing H₂ and organic acid production (45). In glycerol fermentations by *C. butyricum* and C*lostridium pasteurianum* DSM 525, MV and NR, respectively, elevated 1,3-propanediol and butanol yields while decreasing hydrogen production (48, 50). These results demonstrate the role of EMs in inhibiting hydrogen evolution and redirecting electrons from Fd to NADH to promote alcohol synthesis.

In addition to viologens, anthraquinone-2,6-disulfonate (AQDS) and ferrihydrite (Fe(OH)_3_) have been identified as effective, albeit with a lesser promoting effect compared with MV and BV, in enhancing alcohol yields during the fermentation of *Clostridium sp*. BC1 (47). Recently, biochar has emerged as a cost-efficient EEM with high environmental benefits. Biochar has been shown to enhance both ethanol and butanol production in anaerobic fermentation (51, 52), as it can modulate reducing power during fermentation and accelerate the transition from acidogenesis to solventogenesis. Although biochar is less effective than viologens in enhancing alcohol production, it offers advantages in cost and environmental friendliness.

### SCFAs production in anaerobic fermentation

SCFAs, a subset of carboxylic acids containing 2–5 carbon molecules, are the dominant products in acidogenic fermentation, valued for their broad application in food, pharmaceutical, and chemical industries (63, 64). For SCFA production, different bacteria exhibit distinct advantages for specific acids. *Clostridium* species are most widely studied for butyric acid production due to their substrate versatility (5, 30). *Propionibacterium* strains are specialized for propionic acid synthesis, whereas *Acetobacter* and several thermophilic bacteria are effective in acetic acid production (65). Extensive studies have explored various methods to enhance the production of SCFAs in anaerobic fermentation, such as strain adaptation and metabolic engineering. Compared with these strategies, the addition of EEMs has proven to be a more cost-effective approach to accelerate electron transfer, increase bio-redox reaction rates, and promote substrate degradation (66). For instance, Fu et al. reported that adding BV to *C. tyrobutyricum* increased NADH utilization, redirecting carbon flux from acetate production to butyrate biosynthesis, and resulting in a 50% increase in butyrate yield (30). Due to its high redox reversibility, NR can be used by *Methylobacterium extorquens* to reduce CO_2_ to formate, significantly increasing formate accumulation (53). NR also enhances butyrate production by *C. tyrobutyricum* BAS 7 (5). Additionally, quinone components in HS can also function as EMs in fermentation processes. Studies have shown that adding HS in mixed culture dark fermentation can increase both hydrogen and carboxylic acid production (54). Liu et al. found that HA with higher electron-accepting capacity promotes acetate production in the anaerobic fermentation of waste-activated sludge (67). Given the diversity of bacterial strains used in SCFA production, systematic comparative studies are needed to identify the most effective EEMs for specific strain-product combinations, which would help optimize the application of EEMs in SCFA fermentation.

### Hydrogen production

Hydrogen is considered an ideal energy carrier due to its high energy content and clean combustion by-products (44). Anaerobic fermentation is the dominant bioprocess for hydrogen evolution, standing out by its wide feedstock range, high hydrogen yield and productivity, and simple reactor configuration. *Clostridium* species and *Ethanoligenens* are common hydrogen-producing bacteria with high hydrogen yields (6). They express FeFe-hydrogenases that catalyze the reduction of protons to form molecular hydrogen. Although some EEMs such as MV are known to inhibit hydrogen production in favor of the syntheses of alcohols and SCFAs, some studies report that certain EEMs can facilitate fermentative hydrogen production (68). Ye et al. found that the addition of anthrahydroquinone-2,6-disulfonate (AH_2_QDS) to the fermentation of xylose by *C. beijerinckii* NCIMB 8052 shifted electron flow from the butyrate pathway (NADH-dependent) to the acetate pathway (NADH-independent) (55). This reallocation of excess reducing equivalents favored hydrogen production, leading to an increase in hydrogen molar yield by 24%–37%. Atilano-Camino et al. reported that the addition of anthraquinone 2-sulfonate (AQS) during the dark fermentation of glucose by pretreated anaerobic sludge increased hydrogen yield by 11.4% (56). Zhang et al. found that introducing AQS into the fermentation of excess sludge significantly enhanced biohydrogen production, with the yield increasing to 24.9 mL/g VSS (57). Biochar has also been extensively reported to enhance fermentative hydrogen production (6, 44, 58). Unlike other EEMs, however, the mechanisms by which biochar promotes hydrogen production are relatively complex. Biochar facilitates hydrogen generation through various pathways, including cell immobilization, trace element provision, pH buffering, and redox regulation (44). The effectiveness of different EEMs appears to be strain-specific. For instance, although some EEMs like AH_2_QDS enhance hydrogen production in *C. beijerinckii*, others like MV are known to inhibit hydrogen evolution (55, 62). Therefore, careful selection of EEMs based on the specific bacterial strain and metabolic pathway is crucial for optimizing hydrogen yields.

### Electro-fermentation

EF technology combines traditional anaerobic fermentation with electrochemical methods, offering enhanced metabolic control through continuous electron supply (69, 70). Electron transfer in EF mainly relies on extracellular electron transfer (EET). Among these, indirect electron transfer (IET) through EMs between microbes and electrodes is the primary pathway in microbial electrochemical systems. Numerous studies have shown that EEMs such as MV (71, 72), NR (73), and AQDS (74) can facilitate electron transfer and metabolism in EF, enhancing the yield and purity of products like SCFAs and alcohols.

#### SCFAs production in EF

Compared with traditional anaerobic fermentation, the SCFA synthesis in EF, particularly by acetogenic bacteria like *Clostridium autoethanogenum*, is more dependent on the electrons through EET, necessitating the presence of EEM. For instance, it was found that the NR acted as a key electron transporter during EF of *C. autoethanogenum*, increasing acetate and lactate yields (59). Paiano et al. investigated the electro-fermentation of mixed microbial cultures using glucose as the sole carbon source, with the addition of AQDS and NR (75). They found that both mediators exhibited high selectivity for the condensation of acetate and ethanol into butyrate. NR and AQDS facilitated butyrate production even in the absence of externally available glucose, indicating that they can supply the necessary reducing power for microbial cells in substitution of glucose to convert acetate and ethanol into butyrate. Im et al. combined BES with NR, increasing the conversion rate of CO to SCFAs via the Wood-Ljungdahl pathway, thereby enhancing the conversion of CO to VFAs (24).

#### Butanol production in EF

In butanol-producing strains, particularly solventogenic *Clostridia* like *C. pasteurianum* and *C. acetobutylicum*, the addition of EEMs in BES significantly enhanced butanol production. For instance, Utesch et al. reported that NR increased the yield of n-butanol by 33% in *C. pasteurianum* grown on glycerol in a newly developed BES (50). Early studies by Peguin et al. demonstrated that using *C. acetobutylicum* to ferment glucose and utilizing MV as the EEM under a cathode potential of −0.56 V (vs SHE) increased butanol yield by 467% (61). This MV-mediated cathodic electro-fermentation (CEF), which efficiently produces butanol under controlled pH and cathode potential, was further demonstrated in research by Zhang et al. (62). Zhang et al. also developed a CEF + NR system for ABE fermentation, where the synergistic effect between NR and polarized electrodes induced more carbon sources and electrons for butanol synthesis, achieving effective co-production of butanol (60).

### CO_2_ sequestration and conversion

Bioconversion of CO_2_ into high-value products by acetogens like *C. carboxidivorans* holds significant environmental and economic potential, which can also be enhanced by EEMs. For instance, in an electro-enhanced mixotrophic fermentation process capable of converting CO_2_ into alcohols and SCFAs, adding different EEMs, such as biochar and MV, could significantly increase the carbon efficiency and autotrophic CO_2_ fixation (26). Another study reported that electrodes modified with NR and MV improved the production of value-added chemicals and boosted microbial electro-synthesis of biofuel products from CO₂ (76). In pursuit of a sustainable approach to CO₂ sequestration and conversion, researchers have utilized electropolymerized NR (PolyNR) in enzyme fuel cells (EFCs) to enhance the electrochemical performance of enzyme electrodes, facilitating CO₂ reduction to formate and improving formate yield (77).

### Other applications

The electron shuttle property of EEMs leads to their widespread application in redox reactions *in vitro*. Since the late 1960s, MV has been widely applied to determine hydrogenase activity, serving as a reliable indicator of hydrogen generation or utilization capacities (78, 79). EEMs are electrochemically active and widely used in biosensors (80). Modern electrochemical biosensors utilize these mediators to record the oxidative activity of enzyme systems, significantly enhancing the accuracy and reducing errors associated with previous biosensor designs (81). Additionally, the synergistic use of different EEMs can further improve biosensor performance. Compounds such as ferrocene, NR, and methyl blue have been demonstrated to bind to yeast cells, optimizing biochemical biosensor applications for biological oxygen demand (BOD) analysis (82). For example, Wang et al. employed a p-benzoquinone (BQ)-mediated amperometric biosensor for the early detection of wastewater toxicity to activated sludge (83). Li et al. developed a rapid colorimetric biosensor using *Escherichia coli* as a model bacterium and BQ as an electronic mediator, enabling the swift and straightforward detection of Cu and Hg toxicity in water (84). EEMs play an important role in bioremediation. HS, for instance, contributes to pollutant degradation by enhancing microbial redox respiration functions (85). Similarly, MV has been used in bioremediation processes for the removal of pollutants from groundwater (32, 86).

## MECHANISMS OF EEMs

The diverse applications of EEMs across various fermentation scenarios discussed above demonstrate their effectiveness in enhancing the production of value-added products. However, to fully harness their potential and optimize their implementation, it is crucial to understand the underlying mechanisms by which these mediators influence microbial metabolism and electron transfer. This section elucidates these mechanisms, providing the theoretical foundation that explains the enhanced product yields and metabolic shifts observed in the previous applications.

### Elevating NADH levels via inhibiting hydrogenase activity

**Fig 3:**
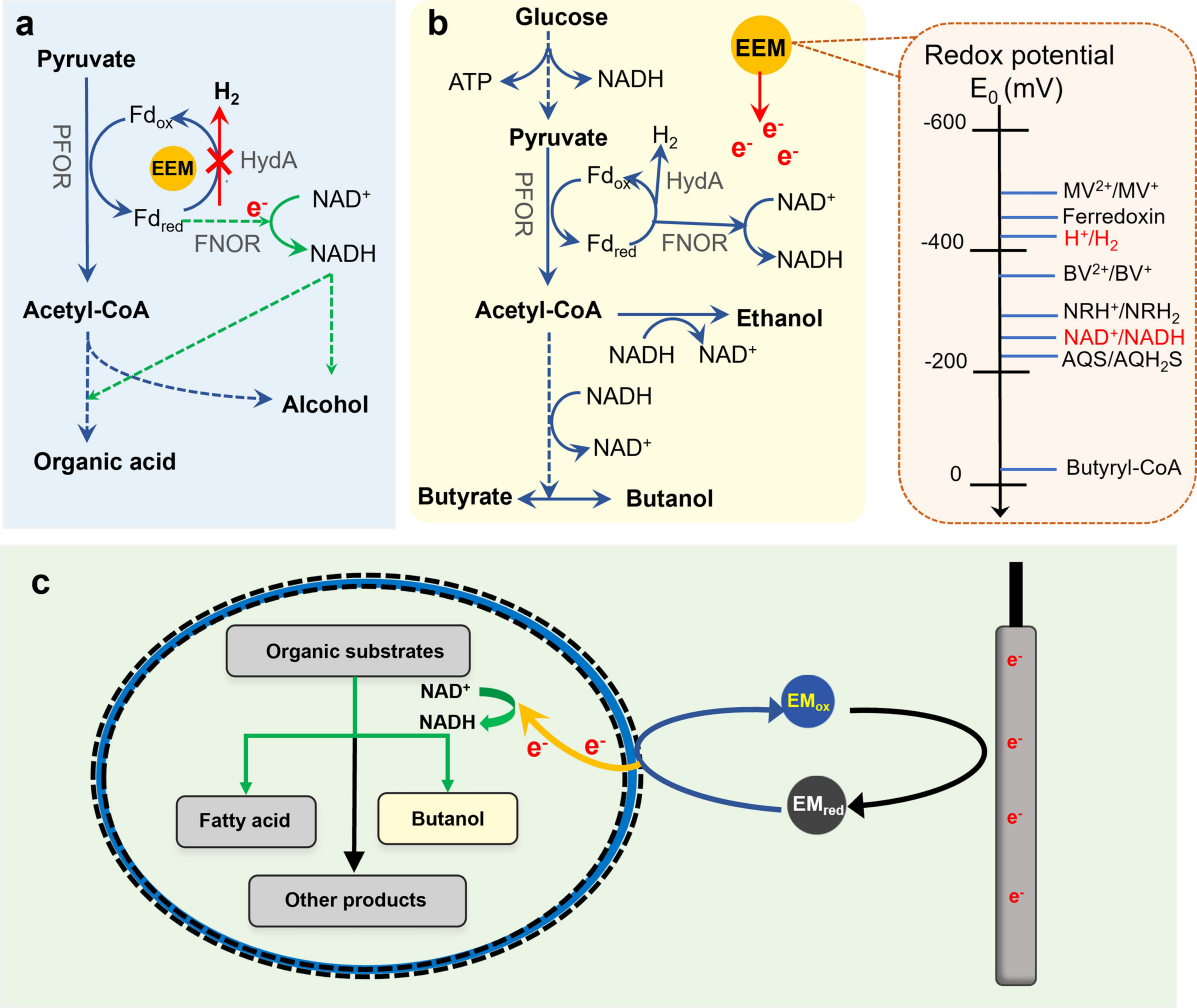
(a) The effect of EEMs on NADH utilization, where the green arrow indicates the enhancing effect of MV and the red arrow indicates inhibition. (b) ORP regulation. (c) EF regulation.

In anaerobic fermentation, the NAD^+^/NADH ratio critically influences the distribution of final products. NADH produced during metabolism must be consumed to maintain redox balance, with its electrons used to form reduced compounds. Hydrogen production is a key process for redox balance in microbes, competing for electrons with other reduced fermentation products, such as alcohol and some SCFAs (8). This process is catalyzed by hydrogenases, which exist in various types based on their location and electron donor/acceptor specificity. In fermentative bacteria, particularly *Clostridium* species, [FeFe]-hydrogenases that interact primarily with Fd are predominant (87). EEMs compete for the active sites of hydrogenase, redirecting electron flow from hydrogen production to NADH generation, thereby enhancing NADH utilization efficiency and achieving high yields and high selectivity of other reduced products (Fig. 3a). For example, when MV is added to the medium, it competes with Fd_red_ for the active site of hydrogenase (88), inhibiting hydrogenase-mediated Fd oxidation. When hydrogen production is inhibited, electrons flow through FNOR, increasing NADH generation (89). This NADH accumulation enhances alcohol synthesis, which serves as the primary pathway for NADH oxidation (49, 88, 90). Beyond hydrogenase inhibition, EEMs can also influence ferredoxin-NADP^+^ reductase activity, further increasing NAD(P)H availability in fermentation processes (91, 92).

### ORP regulation

EEMs are redox-active chemicals that regulate the ORP of the fermentation system and affect energy charge and the NADH/NAD^+^ ratio (93). Common EEMs used in fermentation, such as MV, BV, and NR, have redox potentials of −450 mV, −374 mV, and −325 mV, respectively (94, 95). When EEMs are introduced into the fermentation system, if their ORP is more negative than that of NAD^+^ (−316 mV), they donate electrons to reduce NAD^+^ to NADH (96). The increase in NADH redistributes metabolic flux, promoting solvent production (97–99), as shown in Fig. 3b.

### Acceleration of electron transfer

The electron transfer between electrodes and microbes is essential for production efficiency in EF and other BES. However, many microorganisms are either non-electroactive or inefficient at accepting electrons from the cathode (26). EEMs can act as bridges between electrodes and microbes through redox cycling, enhancing electron transfer and affecting the distribution of carbon and electron flux during fermentation (Fig. 3c). In the presence of EEMs, such as NR and MV, the EF system enhances substrate degradation and induces more carbon flux to alcohol syntheses (68, 100). Electrochemical analyses, such as cyclic voltammetry, have shown that EEMs significantly increase the charge transfer and facilitate EET, maintaining a high current within the system (101). EEMs also facilitate electron transfer from the microbes to the terminal electron acceptors. Numerous studies indicate that the ability of EEMs to penetrate cell membranes is critical for electron transfer to extracellular acceptors. MV, for instance, can only penetrate the outer membrane of microorganisms, restricting its electrochemical activity to the cytoplasm and thereby limiting the potential pathways for MV-mediated EET. In contrast, NR can bind to the cell membrane, thereby more effectively facilitating EET.

### Factors influencing EEMs

The effects of EEMs in fermentations are influenced by multiple factors, including supplementation concentration, timing of addition, fermentation conditions, stability, biocompatibility, and toxicity. This review highlights these critical factors affecting EEM performance and offers guidance on optimizing their use in anaerobic fermentation.

## CONCENTRATION

The effective concentration of EEMs is crucial for optimizing fermentation performance. Both insufficient and excessive mediator levels can negatively impact the process. At low concentrations, the effect of EEMs on enhancing electron transfer and facilitating fermentations may be hindered. For example, in an attempt to employ MV to inhibit butyrate production to promote butanol and ethanol syntheses, an addition of 50 µM MV did not reduce butyrate yields but decreased ethanol production (45). However, at a higher concentration of 500 µM, MV demonstrated its regulatory role by increasing butanol concentration to 11.0 g/L while significantly reducing butyrate production. It is important to note that excessively high concentrations of EEMs can also be harmful. Beyond economic considerations, a high amount of EEM may inhibit metabolic pathways or cause cytotoxicity. Panneerselvam et al. reported that in the fermentation of *Clostridium ragsdalei*, 0.1 mM BV resulted in a substantial decrease in cell concentration and the absence of any liquid products, indicating that the toxicity of BV led to cell death (97).

### Timing of addition

The timing of EEM addition needs to be precisely controlled according to the phase of fermentation and the growth stage of the microorganisms to achieve better effectiveness and cost-efficiency. Solvent production generally occurs during the late stage of cell growth or stationary phase, which influences the optimal timing for EEM addition. Adding EEMs too early or too late can either inhibit cellular metabolism or have decreased effectiveness. Fu et al. investigated the impact of adding 1 µM BV at four distinct phases of fermentation with *C. tyrobutyricum* (30). When BV was added at the beginning of fermentation (0 h), its toxicity inhibited the production of acetate and butyrate. The addition of BV during the early (12 h) or mid-exponential (24 h) phase had a negligible inhibitory effect on cell metabolism and significantly enhanced butyrate production, increasing yields by 23%. However, adding BV during the late exponential and early stationary phases (48 h) had a relatively minor impact on cell metabolism. This study highlights the importance of addition timing, which provides a valuable reference for optimizing EEM application in other fermentation processes.

### Fermentation conditions

Fermentation conditions such as pH, redox potential (ORP), and temperature can affect the chemical state, activity, or stability of EEMs, consequently influencing their regulatory effects. Mediators might be unstable or display altered electron transfer capabilities at specific pH levels. Peguin et al. reported the batch cultivation of *C. acetobutylicum* using a three-electrode potential system with 1 mM MV as an EEM, under controlled pH conditions of 5.0 and 5.5. The study observed that compared with the control, butanol yield increased by 7% at pH 5.0 and by 56% at a pH of 5.5 (61). Similarly, Du et al. found that in MV-mediated fermentation of *C. tyrobutyricum*, MV was more effective at controlling electron flow and metabolic fluxes at pH levels of 6.0 and 6.5 than pH 5.5 and 5.0 (45). ORP refers to the ratio of activity between oxidizers and reducers and is also defined as the tendency of a chemical substance to donate or accept electrons (102), which is an important factor in controlling or monitoring dark fermentation systems. Changes in ORP can also affect the redox state of EEMs, thereby impacting the effectiveness of fermentation regulation. Taking MV as an example, it has been reported that in the potential range of −900 to −650 mV versus Ag/AgCl and at a pH higher than 6, MV primarily exists in the form of MV^+^ (32). Cheng et al. previously found that in electro-enhanced mixotrophic fermentation, the addition of MV in its MV^2+^ form, while maintaining the potential at −757 mV (vs. Ag/AgCl), increased the molar ratios of alcohol/acid, butanol/ethanol, and hexanol/ethanol by 12.3%, 33.7%, and 10.2%, respectively, significantly boosting butanol and hexanol production. In the study, the formation of MV^+^ was confirmed by observing changes in the color of the fermentation broth (i.e., turning blue and then fading), which indicated that MV^+^ was gradually oxidized back to MV^2+^ as it transferred electrons to the cells (26).

### Biocompatibility and toxicity

Many EEMs exhibit toxicity to fermentation bacteria or the environment, necessitating the selection of EEMs with biocompatibility and concentration control. For instance, adding 1 mM MV increased the yields of 1, 3-propanediol and butyrate of *C. butyricum* but significantly reduced biomass growth (49). MV can also cause serious environmental issues due to its toxicity to non-target organisms, persistence, and bioaccumulative potential (103). Quinones, such as hydroquinone, can dissipate membrane potential and increase the permeability and leakage of cellular contents (104). Different EEMs exhibit varied toxicity, which should be carefully compared before use in fermentation. However, a systematic comparison of current EEMs regarding their toxicity to fermentation microbes is still lacking. BV also shows toxicity but is effective at a 10-fold to 100-fold lower concentration than MV and NR in enhancing alcohol production in ABE fermentation (30). Therefore, selecting EEMs with lower toxicity or effective at lower concentrations is a promising approach to mitigate this issue.

### Stability

The stability of EEMs during anaerobic fermentation is essential, as degradation or transformation can compromise their effectiveness, leading to the need for additional supplementation and increased process costs. Different EEMs exhibit varied stabilities, which significantly influence their electron mediation performance (82). Compared with artificial EEMs, natural EEMs such as riboflavin or phenazines secreted by organisms are more likely to be unstable in the long-term fermentation process and BES (105). Therefore, high chemical stability should be a key criterion when selecting EEMs in fermentation for value-added products.

## FUTURE PROSPECTS

Although the rapid development of EEMs in anaerobic fermentation demonstrates great industrial potential through enhanced yield, selectivity, and productivity, cost remains a major challenge. Some EEMs, especially the biogenic ones, can only be used in lab-scale investigations due to their high costs. Artificial EEMs, although much cheaper, still account for a big part of the fermentation cost in industrial scenarios. In some cases, they may affect the purity and quality of fermentation products, necessitating subsequent purification and treatment, which further increases the cost. The second limitation is that the supplementation of EEMs requires more regulation to control its concentration. Studies showed that concentration is a key influencing factor of EEMs that should be optimized, as high concentrations might show an inhibitory effect (5). EEMs can also cause environmental and health issues if released into the environment. For example, MV can disrupt respiratory functions and show high toxicity to aquatic organisms, plants, and soil microorganisms (106–108). MV is also highly toxic to humans if ingested and can lead to death (109). Therefore, careful disposal or recycling of EEMs post-fermentation is essential. Furthermore, some EEMs cannot be recycled after fermentation, lowering their efficiency and increasing the cost. Thus, the development of stable and recyclable EEMs is in need.

Given the limitations of current EEMs, future research could focus on the investigation of more promising EEMs with cost-efficiency, stability, recyclability, and environmental friendliness. More efficient EEMs should be developed to regulate the fermentation network for specific value-added products. Novel materials combined with chemical modification can increase the stability and electron transfer efficiency of EEMs. Another direction is the development of cost-efficient EEMs, which ensures cost control in industrial applications. The environmental impact should also be considered in developing novel EEMs. Biochar is a good example of an environmentally friendly EEM that ensures the sustainability of the fermentation process. In addition, further studies should be conducted to investigate how microbes interact with EEMs and how these interactions affect microbial metabolic pathways and product synthesis. The investigation of the regulation mechanisms can facilitate the development of more efficient EEMs. EEMs can also be integrated with other technologies and processes to maximize the production of value-added products, such as metabolic engineering and photo-fermentation. Moreover, EEMs can be used in pollutant monitoring and removal in different environments (83, 84, 110). Furthermore, systematic comparative studies are needed to evaluate the effectiveness of different EEMs across various strain-product combinations. Current research mainly focuses on individual cases, making it difficult to determine the optimal EEM for specific applications. Such comprehensive studies would help establish guidelines for EEM selection in different fermentation scenarios. These research directions of EEMs not only can propel the advancement of fermentation to produce value-added products but also hold the potential for their application across diverse fields such as energy, environmental protection, food, and industry.

## CONCLUSION

This paper provides a concise review of the characteristics, applications, and underlying mechanisms of EEMs in diverse fermentation processes. It summarizes both commonly used and newly emerging EEMs and introduces various application scenarios, including the production of alcohol, SCFA, and hydrogen. EEMs regulate microbial metabolism via multiple pathways, such as redox regulation, electron transfer acceleration, and enzyme activity manipulation. Furthermore, the paper elucidates the factors that influence the regulatory effect of EEMs, highlighting the necessity for precise control in their addition. Future research could focus on the development of highly efficient EEM while also taking into consideration both cost and environmental impact.

## SUPPLEMENTAL MATERIAL

Fig. S1 aem.00495-25-s0001.docx Representative redox-active groups of typical EEMs.

ASM does not own the copyrights to Supplemental Material that may be linked to, or accessed through, an article. The authors have granted ASM a non-exclusive, world-wide license to publish the Supplemental Material files. Please contact the corresponding author directly for reuse.
