# Supplementary material for: Application of exogenous electron mediator in fermentation to enhance the production of value-added products
Source: Appl Environ Microbiol. 2025 May 12;91(6):e00495-25. doi: 10.1128/aem.00495-25 (PMC12175503; doi:10.1128/aem.00495-25)
Supplement: Fig. S1 — Representative redox-active groups of typical EEMs. [file aem.00495-25-s0001.docx]

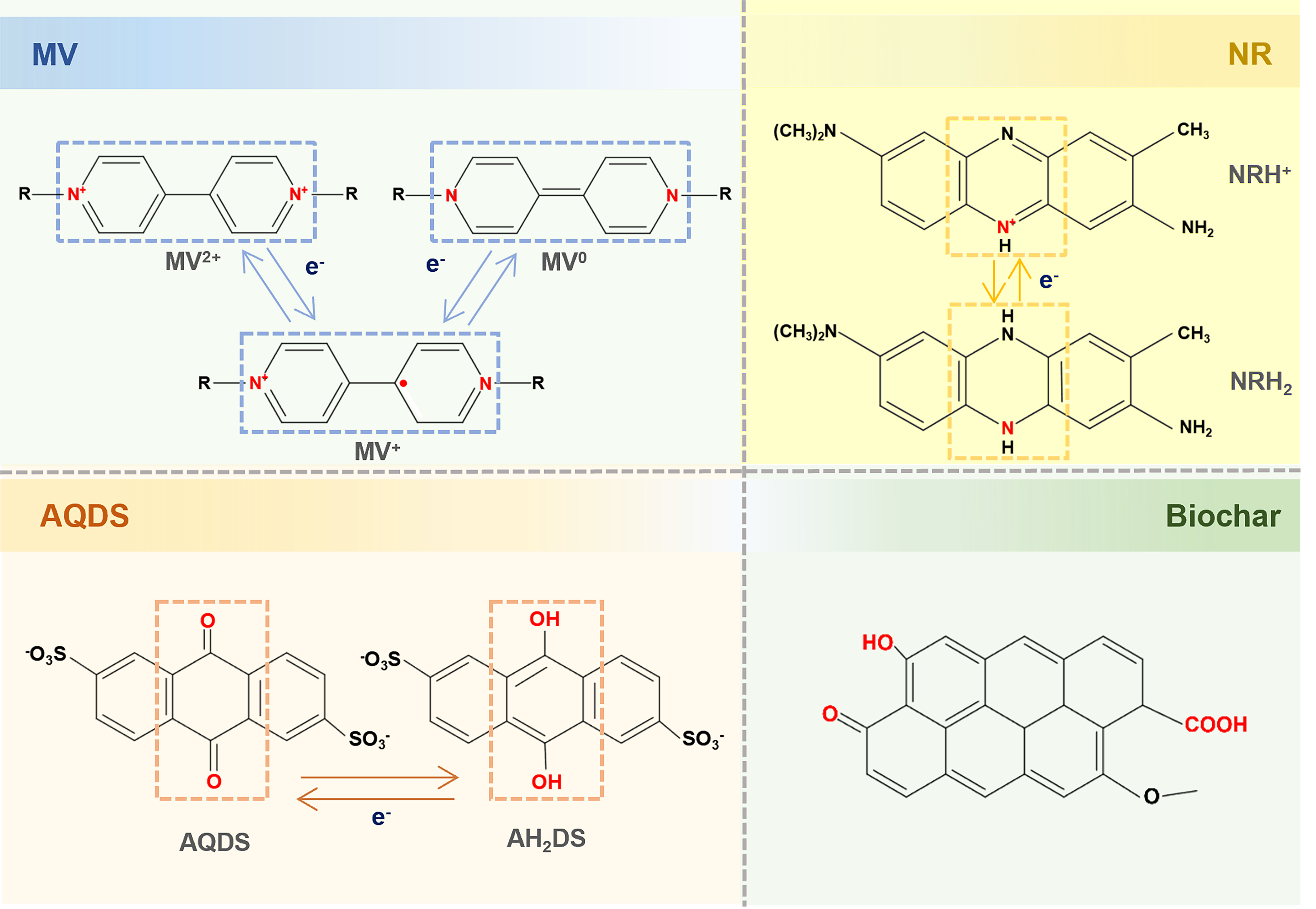


Fig. S1 Representative redox-active groups of typical EEMs: MV, NR, AQDS, and biochar. The redox-active sites of EEMS are highlighted in red.
